# Monthly Summary

**Published:** 1869-05

**Authors:** 


### MONTHLY SUMMARY.

---

*Comminuted Fracture of Nasal Bones and Right Superior Maxilla; Sinking of Eyeball into Maxillary Sinus.*—Dr. Langenbeck gives an account of a railroad official whose head was caught between a locomotive and its tender. The eyelids were torn away from the orbit, and a deep wound ran down from the inner canthus to the upper lip. A probe could be passed into the antrum, not a trace of the eyeball could be be found; while in the orbit was a bluish-black pulsating mass. The nasal bones comminuted. Patient conscious, but sleepy, pulse slow, violent pain on right side of head. A week afterward, as head-symptoms disappeared, and the extravasated blood had been somewhat absorbed, a closer scrutiny could be made. The eyeball was discovered to have escaped from the orbit into the antrum by a hole, in the orbital margin of the upper jaw, big enough to admit the finger easily—the axis of the eye standing vertical, the cornea downward.

The fragments of bone were adjusted as well as possible, and the eyeball replaced in the orbit. It was uninjured, and vision was perfect.

About ten weeks after, by two blepharoplastic operations the eyelids were brought into a tolerably good condition. They could be closed, and usually so remained, but could be opened enough to expose the cornea and permit sight. The globe was however perfectly immovable. About five months after the injury, ulceration and suppuration of the cornea occurred, and the globe atrophied.—*New York Med. Journal.*

*Dentistry in Japan.*—In some interesting notes on the state of Medicine in Japan, Dr. Alex. M. Vedder writes (*Am. Jour. Med. Sci:*) "It might not be amiss, in the course of these remarks, to add a few words concerning a kindred profession to our own. I refer to dentistry. This *trade*, for such it may be more fitly considered in Japan, is carried on by a very low class of people, usually peripatetic in their habits, and who carry with them a box covered with brass ornaments, by which their occupation is recognized. Now, the extraction of a tooth by one of these gentry is regarded by the Japanese as a capital operation, and not without reason, if the information given me be reliable, that death (from tetanus, I presume) is not unfrequently the result. The tooth is extracted by the operators fingers, but not until it has been well loosened by means of a stick and a mallet vigorously wielded. The operation is seldom performed, but I saw some teeth in possession of one of these charlatans that had large portions of the alveolar process attached. In the face of these facts it can scarcely be credited that artificial teeth, sustained by *atmospheric pressure*, have been in use from time immemorial. These teeth are carved out of sea-horse ivory, the molars being plentifully studded with little brass bosses, and the whole stongly mounted upon a base cut from the hard shell of a species of gourd, and carved to conform to the irregularities of the gums and palate. I have several sets of these teeth in my possession; they are not expensive, the very best, a complete upper set, costing about five boos, or about one dollar and sixty cents. Colossal fortunes are not accumulated from dentistry in Japan, as may be inferred from the foregoing.—*Med. Gazette.*

---

*Plaster Impressions.*—H. C. Bartleson in the *Dental Register*, describes his method as follows: I select the cup most suitable and take an impression in wax in the usual manner, only that I make no attempt to get the roof of the mouth perfect, save where the posterior border of the plate is to come, and I draw it in the manner indicated to secure as perfect an impression of the teeth as possible. Then before the wax is cool, I trim it with a warmed knife, cutting off all that projected beyond the cup posteriorly, and shave away to the depth of an eighth of an inch all that which came in contact with the gum and hard palate, except at the posterior border of the impression, when I leave about a line's breadth, (to keep the plaster from running down the throat)and that which is close to the teeth, to preserve their form. The surface thus made I roughen by coarse scratches for the plaster to fasten to. Thus prepared, I re-introduce the now cold impression, to see that it has not been changed, and to observe the best manner it is accommodated to the the teeth. I then pour the plaster where the wax has been removed to the amount that will slightly exceed the quantity of wax which was shaved away, and proceed in the usual manner of taking plaster impressions, pressing it firmly home. All excess of plaster will pass out over the gum where the teeth have been removed. I allow the plaster to become perfectly hard, having no fear of its being held by the teeth, as it does not come in contact with them. This method secures the accuracy of wax around the teeth, combined with the perfection of plaster for the palatine surface and gums.

